# Extracorporeal Membrane Oxygenation (ECMO) Dependent Acute Respiratory Distress Syndrome (ARDS): A Systematic Review and Meta-Analysis

**DOI:** 10.7759/cureus.25696

**Published:** 2022-06-06

**Authors:** Dhan B Shrestha, Yub Raj Sedhai, Pravash Budhathoki, Suman Gaire, Prarthana Subedi, Swojay Maharjan, Mengdan Yuan, Ankush Asija, Waqas Memon

**Affiliations:** 1 Department of Medicine, Mount Sinai Hospital, Chicago, USA; 2 Department of Internal Medicine, Virginia Commonwealth University School of Medicine, Richmond, USA; 3 Department of Internal Medicine, BronxCare Health System, Bronx, USA; 4 Department of Emergency Medicine, Palpa District Hospital, Palpa, NPL; 5 Department of Internal Medicine, Nepalese Army Institute of Health Sciences, Kathmandu, NPL; 6 Internal Medicine, University of Michigan, Ann Arbor, USA; 7 Department of Internal Medicine, West Virginia University School of Medicine, Morgantown, USA

**Keywords:** systematic reviews, meta-analysis, mechanical ventilation, ecmo, ards

## Abstract

Background: Extracorporeal membrane oxygenation (ECMO) has emerged as a newer method for managing severe acute respiratory distress syndrome (ARDS) and ARDS refractory to conventional management. However, its current role in the management of ARDS is not clear. Therefore, we conducted this meta-analysis to compare the mortality rates of ECMO over conventional management in ARDS.

Methods: PubMed, PubMed Central, Embase, and Scopus were searched using appropriate keywords. We selected studies in adults with ARDS that compared the outcomes of patients treated with ECMO vs. conventional management. Cochrane Risk of Bias (RoB) 2.0 and the JBI (Joanna Briggs Institute) quality assessment tools were used for assessing the risk of bias in RCTs and observational studies, respectively. The I^2 ^statistic was used to evaluate heterogeneity, and quantitative synthesis was performed using fixed or random effects to pool studies based on heterogeneities. Meta-analysis was conducted using Revman 5.4.

Result: Twelve studies were included in this meta-analysis. As compared to the conventional management (mechanical ventilation: MV), patients treated with ECMO had lower odds of 30-days mortality (OR, 0.56; 95% CI, 0.37 to 0.84) and 90 days mortality (OR, 0.59; 95% CI, 0.41 to 0.85). However, there was no significant difference between in-hospital mortality (OR, 0.75; 95% CI, 0.40 to 1.41) and intensive care unit (ICU) mortality (OR, 1.00; 95% CI, 0.36 to 2.79). Similarly, length of hospital stays (LOS) (MD, 3.92; 95% CI, -6.26 to 14.11) did not show statistically significant differences across the two groups. However, the average ICU stay (ICU LOS) was 7.28 days longer in the ECMO group compared with the MV group (MD, 7.28; 95% CI, 2.55 to 12.02).

Conclusion: Twenty-eight days and 90-days mortality were decreased in patients managed with ECMO compared with the MV group. Also, ICU LOS was found to be longer in the ECMO group. Furthermore, no statistical difference was found between the two groups for in-hospital mortality and hospital LOS.

## Introduction and background

Acute respiratory distress syndrome (ARDS) is one of the most common presentations in the intensive care unit (ICU). It has been managed conventionally by mechanical ventilation, and lung-protective ventilation has remained a cornerstone of ARDS management. With the discovery of extracorporeal membranous oxygenation (ECMO), it is considered a tool for managing severe ARDS. ECMO is a modified cardiopulmonary bypass circuit that provides gas exchange and ensures systemic perfusion to sustain the patient's life in pulmonary and cardiac failure refractory to conventional therapy. It alleviates the need for high airway pressures, thereby allowing the lungs to rest and prevent the effects of high pressures in the airway [1]. The associated risk of complications related to ECMO in patients with refractory ARDS is found to be coagulopathies, infections, hypoxia, ischemia, multi-organ failures, and others [2]. The two randomized controlled trials (RCT) could not confirm the superiority of the technique over more conventional management [3,4]. However, studies in the recent past show that ECMO and ventilator techniques have better survival rates and have improved six-month disability-free survival [5,6,7]. Given the lack of adequate data that compares the use of ECMO with other modalities of management in refractory ARDS, our study aims to evaluate the overall outcomes and outcome predictors, the etiologies, and the risk factors associated with ECMO dependent ARDS.

## Review

Objectives

To compare mortality and length of hospital and ICU stay in patients with ARDS managed with ECMO to conventional treatment with mechanical ventilation.

To compare serious adverse events among patients with ARDS managed with ECMO to conventional treatment with mechanical ventilation.

Methods

We used the PRISMA guidelines for this metanalysis [8]. The protocol has been registered in the International prospective register of systematic reviews (PROSPERO) (CRD42020215494) [9].

*Eligibility Criteria* 

Types of studies: We included prospective as well as retrospective observational studies and randomized clinical trials, which compared the mortality rate, clinical improvement and recovery, length of hospital stay, adverse effects of ECMO, mean difference of clinical improvement, and healing among patients receiving ECMO for ARDS as compared to conventional/conservative treatment. We have only included the studies after 2000 as there have been significant changes in ECMO management.

We have not included editorials, comments, viewpoint articles, systematic reviews, and meta-analyses. In addition, we have not included studies in which ECMO is used for the management of cases other than ARDS and the studies which have not mentioned our outcome of interest.

Types of participants: We included all patients suffering from ARDS > 18 years of age receiving ECMO or treated with conventional or conservative treatment. We have not included non-ARDS patients, less than 18 years of age, or pregnant patients.

Types of interventions: Interventions included ECMO (extracorporeal membrane oxygenation (venovenous (VV)/venoarterial (VA) or veno arteriovenous (VAV) compared with conventional treatment of mechanical ventilation or other adjunctive therapies. 

Outcomes: We compared mortality at different time durations, cause of death, hospital and ICU length of stay, days on mechanical ventilation, number of days alive, and post-discharge mortality rates between patients receiving ECMO to those receiving conventional treatment with mechanical ventilation. 

Search Methods

Two authors (PB and DBS) independently searched and evaluated the quality of the studies done in the past decade, identified via electronic search in PubMed, PubMed Central, Embase, Scopus, and Google Scholar databases.

Data Collection and Analysis

We extracted the data for quantitative synthesis through Covidence and did the analysis using RevMan5.4 (London, UK) [10,11]. Assessment of heterogeneity was done using the I-squared (I2) test. We used a random/fixed effect for the pooling of selected studies.

Selection of studies: Articles from the literature search were imported to Covidence, and duplicates were removed. Two researchers independently screened the titles and abstracts of all articles included. The conflicts were resolved by discussing with a third reviewer, and articles were finalized for full-text review. The same procedure was used to carry out a full-text review of the screened articles to include in the study. 

Data extraction and management: Two researchers independently extracted data from the included studies, and any discrepancies were resolved through discussion. The extracted data were entered into Revman5.4. We evaluated the quality of studies thoroughly and considered only the outcomes of our interest.

**Figure 1:**
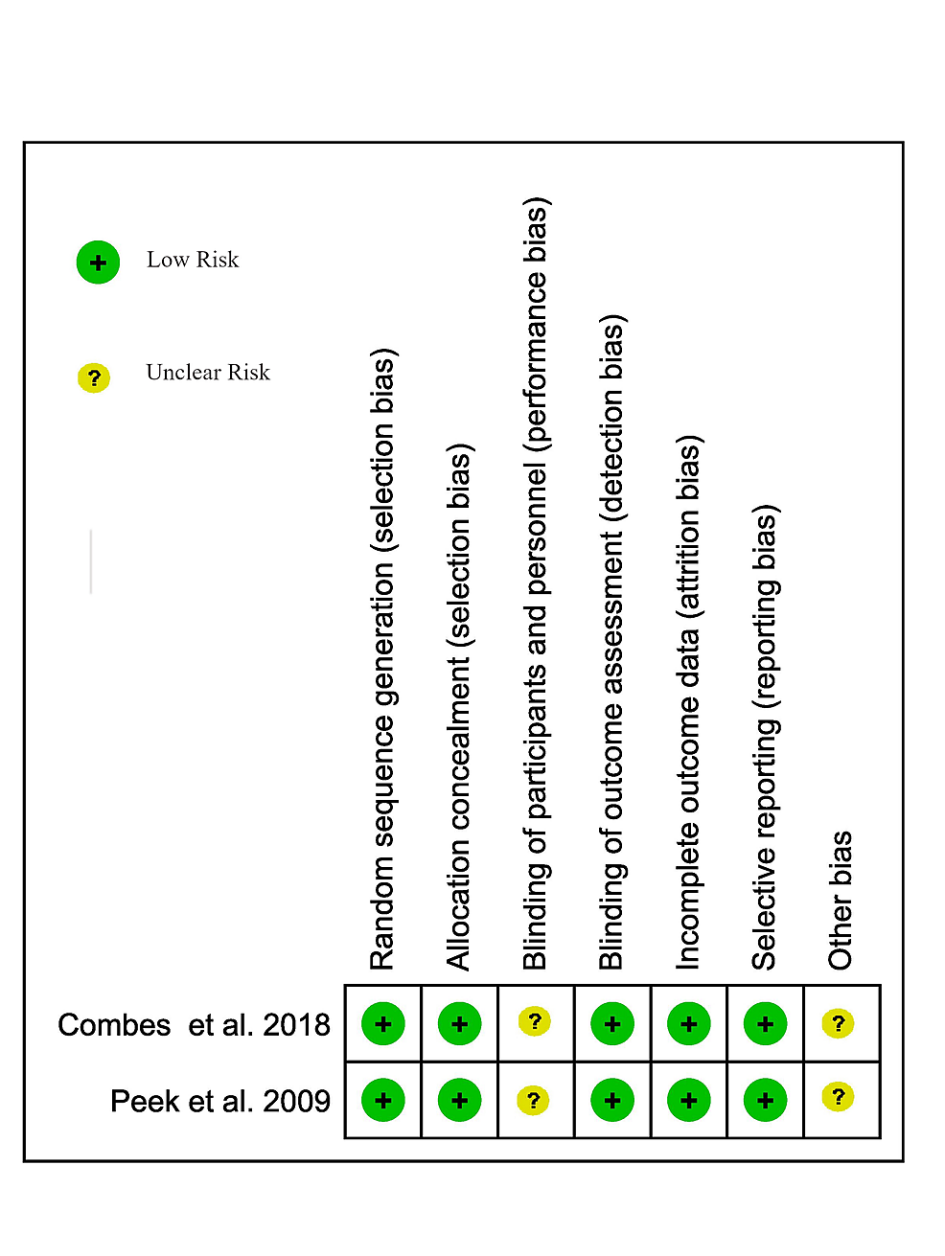
Risk of bias among RCTs using Cochrane ROB 2.0 tool Combes et al. 2018 and Peek et al. 2009 [7].

Assessment of risk of bias in included studies: We used the Cochrane ROB 2.0 tool to analyze our RCTs, and we used the Joanna Briggs Institute (JBI) quality assessment tools to assess the risk of bias in our prospective and retrospective observational studies (Figure 1 and Tables 1, 2) [12,13]. We used RevMan 5.4 for creating a summary of preferences for RCTs using the Cochrane ROB 2.0 tool.

**Table 1:** JBI bias assessment of cohort studies

Questions	Beiderlinden et al. [14], 2006	Bosarge et al. [2], 2016	Wang et al. [15], 2017	Liu et al. [16], 2019
1. Were the criteria for inclusion in the sample clearly defined?	Yes	Yes	Yes	Yes
2. Were the study subjects and the setting described in detail?	Yes	Yes	Yes	Yes
3. Was the exposure measured in a valid and reliable way?	Yes	Yes	Yes	Yes
4. Were objective, standard criteria used for measurement of the condition?	Yes	Yes	Yes	Yes
5. Were confounding factors identified?	No	No	No	No
6. Were strategies to deal with confounding factors stated?	No	No	No	No
7. Were the outcomes measured in a valid and reliable way?	Yes	Yes	Yes	Yes
8. Was appropriate statistical analysis used?	Yes	Yes	Yes	Yes

**Table 2:** JBI bias assessment of case-control studies

For case-control studies	Assanagkornchai et al. [17], 2019	Tsai et al. [18] 2015	Roch et al. [19], 2010	Pham et al. [20], 2013
1. Were the groups comparable other than the presence of disease in cases or the absence of disease in controls?	Yes	Yes	Yes	Yes
2. Were cases and controls matched appropriately?	Yes	Yes	Yes	Yes
3. Were the same criteria used for the identification of cases and controls?	Yes	Yes	Yes	Yes
4. Was exposure measured in a standard, valid and reliable way?	Yes	Yes	Yes	Yes
5. Was exposure measured in the same way for cases and controls?	Yes	Yes	Yes	Yes
6. Were confounding factors identified?	No	No	No	No
7. Were strategies to deal with confounding factors stated?	No	No	No	No
8. Were outcomes assessed in a standard, valid and reliable way for cases and controls?	Yes	Yes	Yes	Yes
9. Was the exposure period of interest long enough to be meaningful?	Yes	Yes	Yes	Yes
10. Was appropriate statistical analysis used?	Yes	Yes	Yes	Yes

Assessment of heterogeneity: The I-squared (I^2^) test was used to assess heterogeneity [21]. We interpreted the I-squared (I^2^) test based on the Cochrane Handbook for Systematic Reviews of Interventions.

Assessment of reporting biases: We assessed the reporting biases through predetermined outcome reporting documentation.

Data synthesis: Statistical analysis was performed using RevMan 5.4 software. Odds ratio (OR) was used to estimate discrete outcomes with a 95% confidence interval (CI). We analyzed the mean differences among the two groups for continuous outcomes using mean and standard deviations when available or after calculating mean and standard deviation when the median, sample size, and interquartile range were reported. Mean and SD was calculated to form median and interquartile range (IQR) using the following estimation for continuous variables (LOHS, ICU LOS) [22].

The fixed/random-effects model was used according to heterogeneities.

Subgroup analysis and investigation of heterogeneity: We presented forest plots to visualize the degree of variation between studies.

Sensitivity analysis: For sensitivity analysis, we examined the effect of the study based on their type (RCT and non-RCT) by excluding non-RCT studies when appropriate and re-running the analysis to find any differences. In addition, non-randomized studies were excluded for sensitivity analysis to find any alterations in the outcomes after removal.

Result

**Figure 2:**
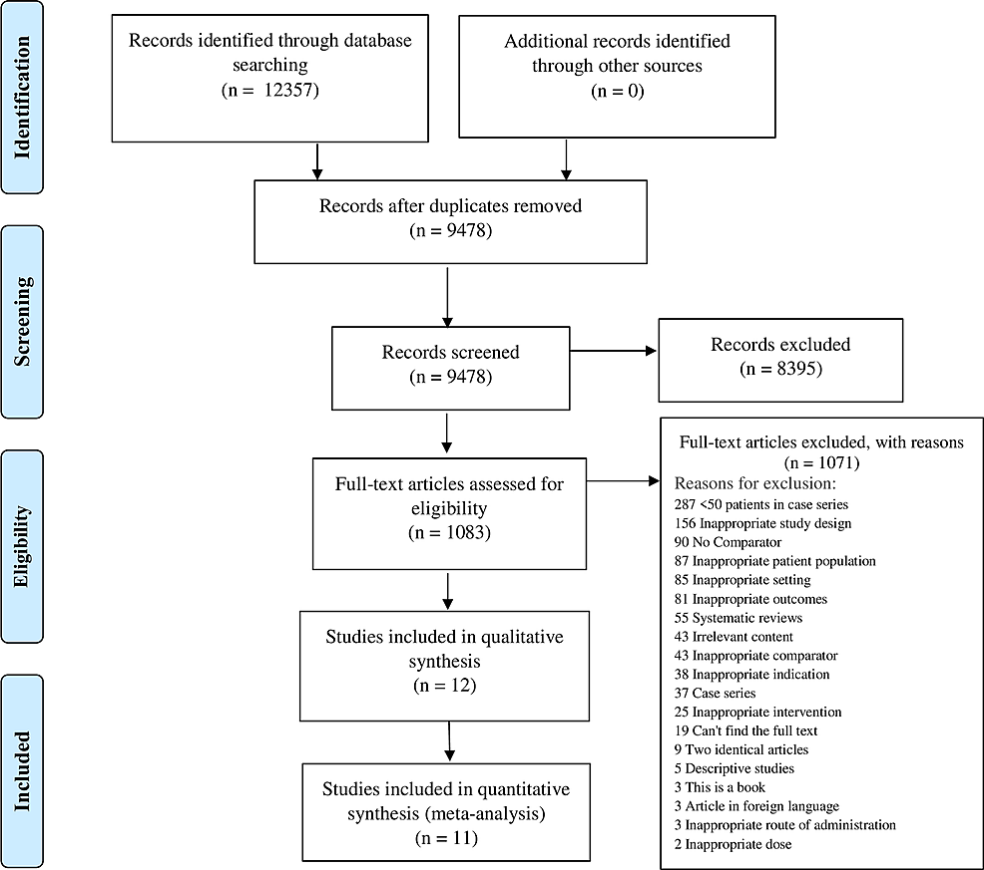
PRISMA Flow Diagram

Twelve thousand three hundred fifty-seven studies were imported from a database search for screening. After removing duplicates, the title and abstracts of 9478 studies were screened. Eight thousand three hundred ninety-five studies were excluded, and full-text eligibility of 1083 studies was assessed. One thousand seventy-one studies were excluded for definite reasons. Twelve studies were included in the quantitative analysis, and 11 were included in the qualitative analysis (Figure 2).

Qualitative Summary

Qualitative details of included studies are presented in Table 3.

**Table 3:** Qualitative summary of included studies ECMO: Extracorporeal membrane oxygenation, APACHEⅡ: acute physiology and chronic health evaluation II, ARDS: Acute respiratory distress syndrome, MV: mechanical ventilation, ICU: intensive care unit, LOS: Length of stay, PEEP: Positive end expiratory pressure, SOFA: Sequential organ failure assessment, SD: Standard deviation, VV: Venovenous, VA: Venoarterial, VAV: Veno arteriovenous, T: Treatment group, C: Control group, IQR: Interquartile range

Study ID	Study type	Population	Intervention	Comparator	Outcomes
Bosarge et al. [2], 2016	Retrospective study	N: 29; T: 15 C: 14 Males; T: 100 % C: 92.9% Median age (median, IQR) T: 40.0 (23.0, 47.0) C: 36.0 (25.0, 47.0)	ECMO (VV/VA /VAV) with adjuncts for ventilator management, including bi-level ventilation, chemical paralysis with cisatracurium, and inhaled nitric oxide	Conventional ventilation with adjuncts, including bi-level ventilation, chemical paralysis with cisatracurium, and inhaled nitric oxide.	Mortality: T: 2/15 C: 9/14 Hospital Length of stay; T: 43.5 (30.0, 93.0), C: 28.0 (14.0, 7.0) Bleeding complications; T: 6/15 C: Not mentioned Thromboembolic complications T: 4/15 C: Not reported
Beiderlinden et al . [14], 2006	Prospective study	N: 150; T: 32 C: 118 Age: T: 42.2±13; C: 41.9±16	Venovenous extracorporeal gas exchange for patient unresponsive to conservative measures.	Conservative treatment with ventilation to keep oxygen saturation >90% with PEEP adjustment, prone position trial, the addition of nitric oxide	Mortality: T:15/32 C:34/118
Tsai et al. [18], 2015	Retrospective case-control study	N: 90; T: 45, C: 45 Age, years: T: 56± 2.4, C:56±2.4 Male: T:32/45, C:34/45 Etiologies of ARDS Infection: T: 30/45, C: 33/45 Pulmonary hemorrhage: T: 5/45, C: 2/45; and so on	In the absence of contraindications: severe pulmonary hypertension (mean pulmonary artery pressure greater than 45 mm Hg or more than 75% of systemic arterial pressure), cardiac dysfunction requiring inotropic support, or history of cardiac arrest or resuscitation; venovenous mode was preferred over venoarterial mode.	Standard ventilation protocols for ARDS were used.	Hospital mortality (among matched) T: 22/45 C:34/45
Roch et al. [19], 2010	Prospective observational study	N: 18; T:9, C:9 Age, median (IQR), years T: 49 (26–57), C: 54 (43–60) Male: T:3/9, C:4/9	ECMO therapy was indicated if patients presented PaO2 to FiO2 ratio of less than 70 mmHg for at least two hours under FiO2 of 1 and PEEP level adjusted to obtain a plateau pressure (Pplat) of 30 cmH2O, or PaO2 to FiO2 ratio of less than 100 mmHg associated with Pplat 35 cmH2O, or respiratory acidosis with pH B7.15 despite respiratory rate C35/min Venovenous ECMO was used. Venoarterial ECMO was used if left ventricular Ejection fraction was <30	Patients were managed with continuous neuromuscular blockade with volume-controlled mechanical ventilation. The tidal volume was maintained at 5-7 ml/kg of predicted weight and PEEP>:10 cm of H2O.	Duration or length of stay, median (IQR), days Mechanical ventilation: T:27 (20–31), C: 12 (8–38) ICU: T:28 (21–33), C:13 (8–48) Hospital: T: 28 (21–40), C:28 (8–50) Mortality: T:5/9, C:5/9 Corticosteroid for ARDS: T: 5/9, C: 3/9 Cause of death: Intractable respiratory failure T: 2/9, C:1/9] Multi-organ failure T: 3/9, C: 4/9 Renal Replacement Therapy At baseline: T:1/9, C:0/9 Day 1: T:4/9, C:0/9 Day 2: T:4/9, C:1/9 Day 3: T:4/9, C:2/9
Assanangkornchai et al. [17], 2019	Retrospective case control study	N:76; T:19, C:57 Age, mean (SD): T:45.9±18; C:55.7±15.2 Male, T:14/19; C:42/57 PaO2/Fio2 ratio, mean (SD) T:56.8 ± 12.9 C:72.9±16.6	16 cases were treated with a venovenous circuit three cases were treated with venoarterial circuit due to refractory hypotension.	Conventional treatment	Mortality in hospital: T:13/19, C:36/57 In ICU: T: 12/19, C:27/57 ICU stay Median, (IQR) in days T:19.7 (12.2, 30.6), C: 7.4 (2.9, 9.9) Hospital stay Median, (IQR) in days T:27.8 (18.1,51.1), C: 16.9 (7.8, 32.8) Continuous Renal Replacement Therapy: T: 10/19, C:15 /57 Bleeding: T: 4/19, C: No mention
Pham et al. [20], 2013	Cohort study and propensity-matched analysis	After matching N: 104; T: 52 C: 52 Age: Mean ± SD: T: 45 ± 13, C: 45± 15 Male: T: 30/52 C: 29/5	Venoarterial and venovenous ECMO in addition to antiviral treatment.	Conventional ventilation treatment without ECMO	Length of MV, days Median (IQR) T: 22 (11.7–35), C: 13.5 (7–21) ICU stay, day Median (IQR) T: 27 (12–52), C: 19.5 (9–26) days Mortality: T: 26 /52, C: 21 /52
Liu et al. [16], 2019	Matched cohort study	N: 171; T: 99, C:72 Age (years): T: 48.6 ± 4.9; C: 50.2 ± 5.3 Male: T: 72/99; C: 52 /72	Extracorporeal membrane oxygenation in addition to conventional treatment.	A conventional lung-protective ventilation strategy was applied. The ventilation settings and hemodynamics were collected. Other treatments were performed routinely by the physician in charge.	Mortality on 28 days: T: 39/99, C: 40 /72 Mortality on 90 days T: 44/99; C: 45 /72 ICU stay (days) (mean ± SD) T: 25.5 ± 18.0; C: 14.8 ± 10.8 Hospital stay (days) (mean ± SD) T: 26.8 ± 19.9; C: 18.6 ± 13.6
Wang et al. [15], 2017	Prospective observational study	N:72; T: 24 C: 48 Male: T: 18/24 C: 33/69 Age in years: T: 38.0± 15.1, C: 44.3± 15.6	ECMO with adjuncts like mechanical ventilation, vasopressors, prone position ventilation use of corticosteroids, muscle relaxants, sedatives, and tracheostomy.	Standard combined therapy is based on the guidelines for the management of ARDS but not ECMO.	MV duration (days) [Median (IQR)] T: 10.0 (6.0, 16.3); C: 9.0 (6.0, 13.0) ICU stay (days): T: 13.0 (9.8, 22.3); C: 11.0 (8.0, 18.0) Hospital stay (days): T: 25.5 (16.5, 31.3); C: 26.0 (15.0, 56.3)
Combes et al. [23], 2018	Randomized controlled trial	N: 249; T:124, C: 125 Age, years: T: 51.9±14.2; C: 54.4±12.7 Male: T: 87/124; C: 90/125	The patient underwent ECMO through percutaneous venovenous cannulation and anticoagulation.	Ventilatory treatment according to increased recruitment strategy, neuromuscular blocking agents, and prone positioning ventilation.	Mortality at day 30: T: 32/124; C: 46/125 At Day 90: T: 46/124; C: 59/125 In ICU: T:44/124; C:57/125 In-hospital: T: 44 /124; C: 57/125 ICU stay, days [median, IQR] T:23 [13–34]; C:18 [8–33] Adverse events Pneumothorax: T: 18/124, C:16/125 Hypothermia (T °< 35°C) T: 28/124, C: 27/125 Hemorrhage requiring transfusion T: 57/124 C: 35/125 Massive hemorrhage (> 10 PRBC) T: 3 /124 C: 1/125
Lei et al [24], 2014	Observational study	N:11, T: 5, C: 6 Males: T: 4/5 C: 4/6 Age (year), [median (Q1,Q3)] T: 73 (46,77), C: 34 (23,46)	ECMO and conventional ventilation	Conventional ventilation	Hospital Mortality T: 1/5 C: 3/6 PaO2/ Fi02 at arrival T: 278±65 mm Hg C: 41±5 mm HG
Shaoyan et al. [25], 2016	Retrospective cohort study	Adult patients with severe ARDS N: 58 T: 28, C: 30 Different parameters like lowest PaO2/FiO2 and pH, the highest PEEP, PaCO2 and serum lactate level, the grade of APACHEⅡ, Murray and SOFA were similar between two groups	ECMO in the treatment group	Conventional treatment in the control group without ECMO	Mortality at 3 Months T: 13/28 C: 17/30 Complications T: 23/28 Bleeding: T: 16/28 GI bleed: T: 5/28
Peek et al [7], 2009	Randomized controlled trial	N: 180; T:90; C:90 Male :T:51/90 ; C:53/90 Age ,yrs(mean±sd) T:39.9±13.4 C:40.4±13.4	ECMO in venovenous mode with percutaneous cannulation.	Conventional management with low volume low-pressure ventilation strategy	Mortality ≤6 months or before discharge T:33/90 C:45/90 Length of hospital stay, days, median (IQR) T:35·0 (15·6–74·0) C:17·0 (4·8–45·3) Severe disability T:0/90 C:1/90

Quantitative Analysis

Eleven studies were included in the quantitative synthesis.

Mortality

**Figure 3:**
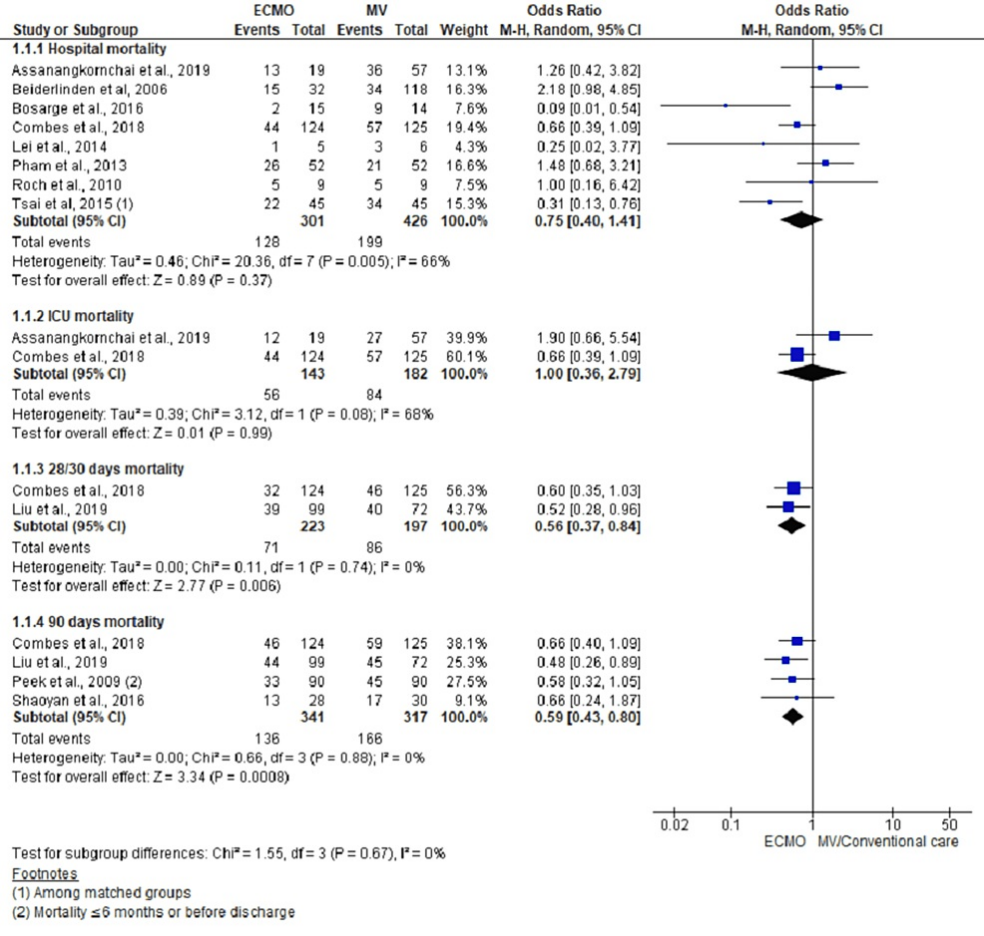
Forest plot depicting mortality outcome comparing ECMO with MV using a random-effect model Subpanel 1.1.1 denotes overall hospital mortality reported in the study; subpanel 1.1.2 denotes mortality during ICU stay; subpanel 1.1.3 denotes mortality within four weeks/a month as reported in the studies, and subpanel 1.1.4 denotes total of 90 days mortality. These counts may overlap with each other, so while pooling, only subtotal is shown in the forest plot. Cited studies are [2,7,14,16-20,23-25].

Mortality in hospital/during study period was reported in eight studies. Pooling their data using random effect model showed no significant reduction in hospital mortality with the use of ECMO over MV (OR, 0.75; 95% CI, 0.40 to 1.41; n= 727; I^2^ = 66%). Similarly, pooling data on ICU mortality from two studies reporting it also did not show significant differences between ECMO and MV (OR, 1.00; 95% CI, 0.36 to 2.79; n= 325; I^2^ = 68%). However, pooling data from two studies on mortality in 28/30 days showed 44% lower odds of event in ECMO group than MV (OR, 0.56; 95% CI, 0.37 to 0.84; n= 420; I^2 ^= 0%). Similarly, 41% lower odds of mortality in 90 days was noted among ECMO group on pooling data from three studies reporting 90-day mortality (OR, 0.59; 95% CI, 0.43 to 0.80; n= 658; I^2^ = 0%) (Figure 3).

Length of Stay

**Figure 4:**
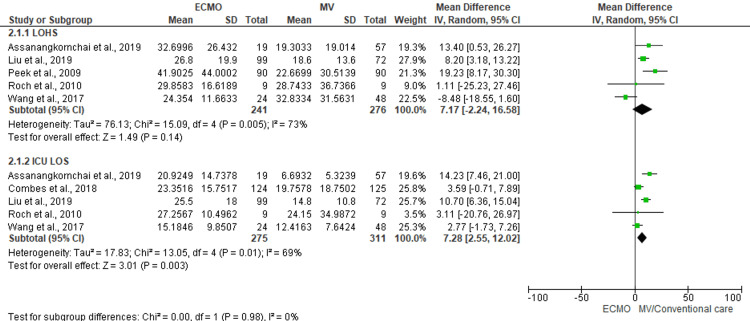
Forest plot depicting the length of stay outcome comparing ECMO with MV using a random-effect model Subpanel 2.1.1 denotes the average length of hospital stay, and subpanel 2.1.2 denotes the average ICU length of stay. Cited studies are [7,15-17,19,23].

Hospital length of stay was reported in four studies. Pooling of the data from reported studies using random effect could not show significant reduction in hospital length of stay (MD, 7.17; 95% CI, -2.24 to 16.58; n= 517; I^2^ = 73%). However, average length of ICU stay was 7.28 days longer in ECMO group comparing with MV group (MD, 7.28; 95% CI, 2.55 to 12.02; n= 586; I^2^ = 69%) (Figure 4).

Discussion

We did not find any significant difference in in-hospital mortality and ICU mortality. A similar conclusion was found for in-hospital mortality in a meta-analysis pooled with one RCT and two observational studies [26]. However, in the same study, the in-hospital mortality reduction was seen with ECMO when results of observational studies were analyzed using a propensity score matching with replacement [26]. However, multiple meta-analyses which have reported 30 days mortality [1], 60-day mortality [1], or 90 days mortality [27], have found lower mortality rates with ECMO than that with conventional ventilation. This is similar to our outcome. A recent meta-analysis [28] had not seen any difference in mortality at 30 days with ECMO or conventional treatment, which had included studies before 2000 AD when the management of ARDS was different from that of recent times, and ARDS management now is evolved a lot in comparison to earlier days with modern technologies. This could have led to the difference in our findings.

We did not find any significant difference in length of hospital stay, but ICU stay was longer in the ECMO group, which is expected. The previous meta-analysis by Mendes et al. [29] has also noted an increase in ICU and hospital length of stay in patients treated with ECMO, which the authors have attributed to increased survival among the patients as compared to the patients treated conventionally. However, in our analysis of the length of hospital stay, Wang et al. [15] have reported the duration only for the survivors in both the cases of ECMO and non-ECMO groups, which could have possibly obscured any difference that could be attributed to increased survival and led to the result. 

Because of the lack of reporting of adverse events, we could not analyze the difference in complications of ECMO. A few studies reported hemorrhagic complications in patients treated with ECMO but not for patients managed with conventional ventilation [2,17]. ECMO to Rescue Lung Injury in Severe ARDS (EOLIA) trial [23] reported an increased incidence of thrombocytopenia and bleeding events requiring transfusion in the ECMO arm compared to the control arm. In a systematic review, bleeding complications were seen in 29.3% of patients treated with ECMO with significant bleeding in 10.4%; the majority causes of bleeding were cannula bleeding (9.3%) [30]. Two studies included in our study reported the need for renal replacement therapy (RRT) [17,19]. Both of them reported increased requirements of RRT in patients treated with ECMO. However, in a recent meta-analysis, management with ECMO was not associated with an increase in RRT incidence [28]. 

We have included the two RCTs that have compared ECMO vs. conventional that have been done in the last two decades [7,23]. Furthermore, we have included other prospective and retrospective studies without any randomization. For example, in their combined meta-regression model, Vaquer S et al. [30] have shown an association between MV duration before ECMO support with mortality. However, in our meta-analysis, the time of the start of ECMO after mechanical ventilation is variable in the studies. Similarly, patients with a PAO2/FIO2 ratio of less than 150 have higher ventilator-free days; the effect was not seen in patients with a higher PAO2/FIO2 ratio [31]. However, there is considerable variation in the studies included in our metaanalysis in terms of the PaO2/Fio2 ratio. Also, there is venoarterial ECMO in selected patients in some studies. Similarly, there might have been wide variation in methods while managing patients with conventional management, including applying for prone positions, using concurrent steroids, and maintaining pressure and volume while managing patients. This could have introduced some bias in our study. 

There have only been two RCTs in the last two decades that have studied ECMO vs. conventional management in ARDS. CESAR [7] showed a mortality benefit of ECMO while EOLIA [23] did not. However, both of them have their limitations. The meta-analyses that have pooled data only from these two RCTs have shown mortality and other benefits of ECMO in ARDS [27,29]. Thus, concerning all these studies and findings, it seems evident that patients benefit from ECMO in ARDS. However, the selection criteria for patients who will benefit is well defined, nor the appropriate time for initiation of ECMO in the patients is evident at present. Further studies should be conducted to shed light on these issues. Our meta-analysis pooled available data; however, a limited number of available papers and heterogenous population and variation in the individual studies are significant limitations of our meta-analysis.

## Conclusions

We analyzed 12 studies in our study. We found lower odds of mortality at 28 days and 90 days in the ECMO group of patients compared with the MV group. Also, ICU LOS was found to be longer in the ECMO group. Furthermore, no statistical difference was found between in-hospital mortality and hospital LOS across the two groups. We could have limited exploration in the analysis due to the limited information available and relatively few studies. Further studies should be conducted to evaluate the outcomes of ECMO in ARDS.

## References

[1] Munshi L, Walkey A, Goligher E, Pham T, Uleryk EM, Fan E (2019). Venovenous extracorporeal membrane oxygenation for acute respiratory distress syndrome: a systematic review and meta-analysis. The Lancet Respiratory Medicine.

[2] Bosarge PL, Raff LA, McGwin G Jr, Carroll SL, Bellot SC, Diaz-Guzman E, Kerby JD (2016). Early initiation of extracorporeal membrane oxygenation improves survival in adult trauma patients with severe adult respiratory distress syndrome. J Trauma Acute Care Surg.

[3] Zapol WM (1979). Extracorporeal membrane oxygenation in severe acute respiratory failure. A randomized prospective study. JAMA: The Journal of the American Medical Association.

[4] Morris AH, Wallace CJ, Menlove RL (1994). Randomized clinical trial of pressure-controlled inverse ratio ventilation and extracorporeal CO2 removal for adult respiratory distress syndrome. Am J Respir Crit Care Med.

[5] Lindén V, Palmér K, Reinhard J, Westman R, Ehrén H, Granholm T, Frenckner B (2000). High survival in adult patients with acute respiratory distress syndrome treated by extracorporeal membrane oxygenation, minimal sedation, and pressure supported ventilation. Intensive Care Med.

[6] Hemmila MR, Rowe SA, Boules TN (2004). Extracorporeal life support for severe acute respiratory distress syndrome in adults. Ann Surg.

[7] Peek GJ, Mugford M, Tiruvoipati R (2009). Efficacy and economic assessment of conventional ventilatory support versus extracorporeal membrane oxygenation for severe adult respiratory failure (CESAR): a multicentre randomised controlled trial. The Lancet.

[8] Liberati A, Altman DG, Tetzlaff J (2009). The PRISMA statement for reporting systematic reviews and meta-analyses of studies that evaluate healthcare interventions: explanation and elaboration. BMJ.

[9] Budhathoki P, Shrestha DB, Dawadi P, Subedi P, Maharjan S, Sedhai YR (2020). Epidemiology of ECMO dependent ARDS, its risk factors and outcome: a systematic review and meta-analysis. PROSPERO.

[10] (2022). Covidence: How can I cite Covidence?. https://support.covidence.org/help/how-can-i-cite-covidence.

[11] (2021). RMW Knowledge Base: Cite RevMan Web in a reference list. https://documentation.cochrane.org/revman-kb/studies-and-references/cite-revman-web-in-a-reference-list..

[12] Sterne JA, Savović J, Page MJ (2019). RoB 2: a revised tool for assessing risk of bias in randomised trials. BMJ.

[13] (2022). Joanna Briggs Institute: Critical appraisal tools. https://jbi.global/critical-appraisal-tools.

[14] Beiderlinden M, Eikermann M, Boes T, Breitfeld C, Peters J (2006). Treatment of severe acute respiratory distress syndrome: role of extracorporeal gas exchange. Intensive Care Med.

[15] Wang ZY, Li T, Wang CT, Xu L, Gao XJ (2017). Assessment of 1-year outcomes in survivors of severe acute respiratory distress syndrome receiving extracorporeal membrane oxygenation or mechanical ventilation: A prospective observational study. Chin Med J (Engl).

[16] Liu SQ, Huang YZ, Pan C (2019). Venovenous extra-corporeal membrane oxygenation for severe acute respiratory distress syndrome: a matched cohort study. Chin Med J (Engl).

[17] Assanangkornchai N, Vichitkunakorn P, Bhurayanontachai R (2019). Characteristics and outcomes of severe ARDS patients receiving ECMO in Southern Thailand. Clin Med Insights Circ Respir Pulm Med.

[18] Tsai HC, Chang CH, Tsai FC (2015). Acute respiratory distress syndrome with and without extracorporeal membrane oxygenation: a score matched study. Ann Thorac Surg.

[19] Roch A, Lepaul-Ercole R, Grisoli D (2010). Extracorporeal membrane oxygenation for severe influenza A (H1N1) acute respiratory distress syndrome: a prospective observational comparative study. Intensive Care Med.

[20] Pham T, Combes A, Rozé H (2013). Extracorporeal membrane oxygenation for pandemic influenza A(H1N1)-induced acute respiratory distress syndrome: a cohort study and propensity-matched analysis. Am J Respir Crit Care Med.

[21] (2022). Cochrane: Identifying and measuring heterogeneity. http://1.cochrane.org/chapter_9/9_5_2_identifying_and_measuring_heterogeneity.html.

[22] (2021). Wayback Machine: Mean variance estimation. https://web.archive.org/web/20181224162602/http://www.comp.hkbu.edu.hk/~xwan/median2mean.html.

[23] Combes A, Hajage D, Capellier G (2018). Extracorporeal membrane oxygenation for severe acute respiratory distress syndrome. N Engl J Med.

[24] Xu L, Wang Z, Li T (2014). Comparison of extracorporeal membrane oxygenation and mechanical ventilation for inter-hospital transport of severe acute respiratory distress syndrome patients. Zhonghua Wei Zhong Bing Ji Jiu Yi Xue.

[25] Qi SY, Wang WT, Chu ZD, Chen CY, Zhou MK, Ren YX, Liu XJ (2016). The clinical analysis of extracorporeal membrane oxygenation for adult severe acute respiratory distress syndrome. Zhonghua Jie He He Hu Xi Za Zhi.

[26] Zampieri FG, Mendes PV, Ranzani OT, Taniguchi LU, Pontes Azevedo LC, Vieira Costa EL, Park M (2013). Extracorporeal membrane oxygenation for severe respiratory failure in adult patients: a systematic review and meta-analysis of current evidence. J Crit Care.

[27] Combes A, Peek GJ, Hajage D (2020). ECMO for severe ARDS: systematic review and individual patient data meta-analysis. Intensive Care Med.

[28] Wang J, Wang Y, Wang T, Xing X, Zhang G (2021). Is extracorporeal membrane oxygenation the standard care for acute respiratory distress syndrome: a systematic review and meta-analysis. Heart Lung Circ.

[29] Mendes PV, Melro LM, Li HY (2019). Extracorporeal membrane oxygenation for severe acute respiratory distress syndrome in adult patients: a systematic review and meta-analysis. Rev Bras Ter Intensiva.

[30] Vaquer S, de Haro C, Peruga P, Oliva JC, Artigas A (2017). Systematic review and meta-analysis of complications and mortality of veno-venous extracorporeal membrane oxygenation for refractory acute respiratory distress syndrome. Ann Intensive Care.

[31] Karagiannidis C, Kluge S, Strassmann S, Windisch W (2016). Extracorporeal carbon dioxide removal. ERS Monograph.

